# Computer-Based Screening of Functional Conformers of Proteins

**DOI:** 10.1371/journal.pcbi.1000009

**Published:** 2008-02-29

**Authors:** Héctor Marlosti Montiel Molina, César Millán-Pacheco, Nina Pastor, Gabriel del Rio

**Affiliations:** 1 Departamento de Bioquímica, Instituto de Fisiologia Celular, Universidad Nacional Autonoma de Mexico, Mexico City, Mexico; 2 Departamento de Bioquimica y Biologia Molecular, Facultad de Ciencias, Universidad Autonoma del Estado de Morelos, Morelos, Mexico; University of Houston, United States of America

## Abstract

A long-standing goal in biology is to establish the link between function, structure, and dynamics of proteins. Considering that protein function at the molecular level is understood by the ability of proteins to bind to other molecules, the limited structural data of proteins in association with other bio-molecules represents a major hurdle to understanding protein function at the structural level. Recent reports show that protein function can be linked to protein structure and dynamics through network centrality analysis, suggesting that the structures of proteins bound to natural ligands may be inferred computationally. In the present work, a new method is described to discriminate protein conformations relevant to the specific recognition of a ligand. The method relies on a scoring system that matches critical residues with central residues in different structures of a given protein. Central residues are the most traversed residues with the same frequency in networks derived from protein structures. We tested our method in a set of 24 different proteins and more than 260,000 structures of these in the absence of a ligand or bound to it. To illustrate the usefulness of our method in the study of the structure/dynamics/function relationship of proteins, we analyzed mutants of the yeast TATA-binding protein with impaired DNA binding. Our results indicate that critical residues for an interaction are preferentially found as central residues of protein structures in complex with a ligand. Thus, our scoring system effectively distinguishes protein conformations relevant to the function of interest.

## Author Summary

Proteins participate in most of the doings of the cells through a variety of interactions. There is an intimate relationship between the function of a protein and its three-dimensional structure, but understanding this relationship remains an unsolved problem, in part due to the limited information on protein structures bound to other biological molecules. On the other hand, thousands of protein structures in the unbound or free form, are made public every year and these differ from those of the bound structures. How to predict the protein structure in the bound form may assist researchers in understanding the structure/function relationship. Here we report that protein structures bound to other molecules tend to present, as central amino acids, those that are critical for binding other molecules. This feature allowed us to identify the protein structures known to be involved in protein interactions from a screening of thousands of structures derived from the free form.

## Introduction

Proteins are dynamic molecules that adopt multiple structures in vitro and in vivo [1]. To study the role protein dynamics has in protein function, a combination of approaches has been used [1]–[6]. For instance, crystallographic structures of proteins associated with different substrate's analogues have been instrumental in understanding enzymatic function [6]. More recently, the role of protein dynamics in the dihydrofolate reductase function has been analyzed using nuclear magnetic resonance relaxation dispersion [5]. Furthermore, techniques such as NMR, hydrogen-deuterium exchange and mutagenesis experiments have provided insights at specific time-scales of protein dynamics and function [7],[8]; however, the detailed understanding of protein dynamics usually requires information over a broad range of time-scales. Thus, computational modeling is becoming central in studying the link between protein dynamics and protein function for multiple time-scales [8].

To effectively link protein dynamics to protein structure and function using computational modeling techniques, it is required to know the structure of a protein bound to a natural ligand, considering that protein function at the molecular level is understood by the ability of proteins to bind to other molecules (e.g., biological macromolecules and/or small molecules). However, public databases of protein structures scarcely show this information: for instance, in September 4 2007, the PDB release contained 45,632 entries including 1,856 protein-DNA complexes (data obtained from the Protein Data Bank [9]), and 1,700 protein-protein complexes (PINT database [10]). Thus, a computational procedure to identify functional conformations of proteins will facilitate the modeling of protein function in terms of protein structure and dynamics.

In this work, we introduce a computational approach aimed at identifying functional conformers of proteins. To explain the basis of our approach, we have established some definitions and axioms.

*Definitions:*

D1: We refer to a **protein function** as a *process* (group of events over time) that depends on the intra and inter molecular interactions of proteins.
D2: A **protein conformer** is the three-dimensional structure of a protein at a given time, and it corresponds to a local minimum in the free energy surface.
D3: A **functional conformer** of a protein is a protein structure that at a given time participates in a particular protein function (e.g., catalysis).
D4: **Critical residues** for a protein function are those residues that upon mutation abolish the activity of the protein. This definition depends on the way the activity was experimentally measured; hence, a (experimentally determined) critical residue may be either a residue critical for maintaining the protein structure or a residue critical for the interaction with other molecules, or both. For the proteins analyzed here, residues that did not tolerate more than 2 substitutions without loosing full activity in vivo were considered critical residues. Here we simply refer to these residues as *critical residues*, unless otherwise specified (i.e., critical residues for ligand binding).
D5: **Central residues** are the most traversed residues with the same frequency in networks derived from a given protein conformation (see Methods and [11,12]). The most traversed residues are identified by an automatic procedure [12] and usually involve 20% or less of the residues in a protein conformer.

Furthermore, to model protein function in terms of protein dynamics, we will assume as *axioms*:

A1: Proteins accomplish their function through a set of conformations
A2: Critical residues for protein function play their roles in that set of conformations.

Note that experimental evidence supports axiom **A1** [1]–[6], but no evidence exists for axiom **A2**. However, if axiom **A2** is correct, we should be able to identify functional conformers of proteins by identifying those conformers harboring preferentially the critical residues for ligand binding.

In order to relate different conformations with different critical residues we need to estimate a property of the residues that varies with the conformation of proteins; the property used in this study is centrality. One of the reasons to choose centrality comes from the observed alteration in the centrality values of critical residues involved in binding in the dihydrofolate reductase enzyme upon ligand binding [13]. Our method scores for the presence of critical residues as central residues in different protein conformers, thus the conformers with higher scores are postulated to be the conformations associated to the interaction of interest.

It is important to note that many possible conformations could be involved in binding a ligand, provided that the ligand as well presents several conformations accessible to the protein. In this regard, our method does not attempt to identify all of them or a specific one. Instead, here we show that our method can determine from a population of protein conformations, which ones are those related to the binding of a ligand.

In summary, the goal of our work is to identify the functional conformers of proteins. For that, we describe a method that accounts for the presence of critical residues important for ligand binding in different protein conformations. We tested our method in 24 different proteins and more than 260,000 conformations of these proteins both in the absence of a ligand or bound to a ligand. Our results indicate that functional conformers harbor preferentially the critical residues for ligand binding as central residues, thus providing a procedure to effectively identify the functional conformers of proteins.

## Results

### Mapping Critical Residues for Protein Function onto Multiple Protein Conformers

Our group [11],[12] and others [14],[15] have previously reported that network centrality is related to the function of the protein. In most of these previous works, every function of the protein (e.g., folding, catalysis) was limited to the analysis of a single protein structure. Considering axiom **A1** (an ensemble of protein conformations accomplishes protein function), the analysis of a single protein structure may not be appropriate to effectively understand protein function. Thus, a procedure that uses multiple protein conformers to identify critical residues may be more reliable.

A first step in our approach is to build a network representation of a *protein conformer* (two residues were linked if they have at least one pair of atoms at 5 Å or less, see Methods). From this network, we determine the central residues as those with the largest transitivity value and the same frequency of occurrence in the network (see Figure S1). The transitivity values were obtained by counting the number of times a residue was in the shortest paths connecting every pair of residues in the network (see Methods). This may be extended to include as many protein conformers as required. In order to estimate the reliability of our procedure to link critical residues with central ones, we used two parameters: sensitivity and specificity. Sensitivity accounts for the fraction of truly predicted critical residues, and specificity for the fraction of truly predicted non-critical residues (see Methods).

**Figure 1:**
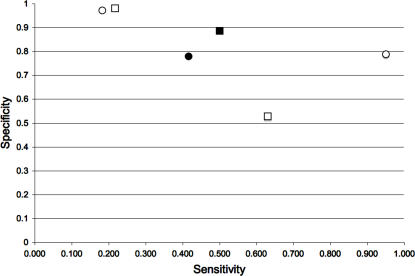
Residue centrality as a marker for protein conformational diversity. The sensitivity and specificity for predicting critical residues are plotted for 2 well-characterized proteins: HIV-protease (squares) and the T4 lysozyme (circles). The empty symbols correspond to the values obtained with a single protein conformer and the shadowed symbols correspond to those obtained with multiple conformers. For comparison, the filled symbols correspond to the values obtained with conserved residues predicted as critical residues (see Methods).

**Figure 2:**
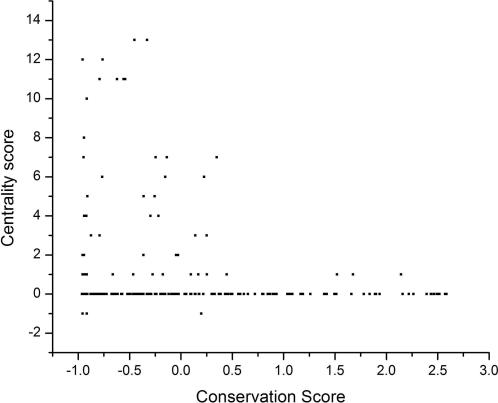
Reconstructing functional and phylogenetic relationships from central residues. For every structure of the SCOP structural family 51351 (Triose Phosphate Isomerase family, including: 1TIM, 1AMK, 1CI1, 1HG3, 1M6J, 1B9B, 1TCD, 1TRE, 1YYA, 1HTI, 1R2R, 1MO0, 1YDV, 1YPI, 1WYI, 8TIM), we calculated their central residues. Using a multiple sequence alignment, we mapped each central residue into the 1TIM structure. Then, we counted the frequency that each position of 1TIM was found as a central residue in all the family (centrality score). Here, we show the relationship of this frequency with a conservation score for each position of 1TIM derived using the Bayesian ConSeq procedure [50]. In this Bayesian approach, the highly conserved positions are those with negative scores.

To this end, we have reported that using multiple protein conformations derived from the normal modes of vibration improves the sensitivity of predictions based on the transitivity [12]. Here, we extend these results for two well-characterized proteins in terms of structure and function, HIV protease [6],[16] and T4 lysozyme [17]. We observed that including a large number of experimentally determined protein conformers improved the reliability for predicting critical residues from the residue's transitivity parameter (see Figure 1). Additionally, we looked at the triosephosphate isomerases (TIMs), a family of enzymes involved in central metabolism. This family includes 16 protein orthologs with known three-dimensional structures in the current PDB release. We observed that central residues shared by most TIM structures, actually correspond to the most conserved residues (see Figure 2).

Thus, including multiple protein conformers does improve the relationship between central residues and critical residues providing support to axiom **A1**: this improvement could be explained by the presence of different central residues in different protein conformations, which is the basis for the contention that a collection of structures corresponds to the functional conformation of the protein.

### Different Sets of Protein Conformers Have Different Sets of Central and Critical Residues

Our results suggest that different sets of protein conformers harbor different sets of central and critical residues. That is, each protein conformer presents several and different central residues. If this were correct, then it would be possible to find the set of protein conformers harboring the critical residues for ligand binding: the functional conformers. That is the contention of axiom **A2**.

**Figure 3:**
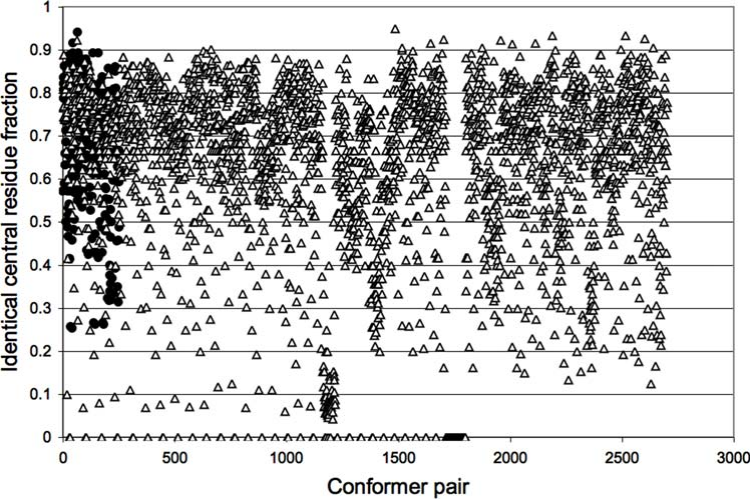
Paired comparison of central residues in protein conformers. The fraction of identical central residues shared by every pair of conformers (y-axis) is plotted against every pair of conformer analyzed (x-axis). The results are shown for every pair between the 23 T4 Lysozyme structures analyzed (filled circles) and the 31 complexed HIV-1 protease structures compared against all the 42 non-complexed HIV-1 protease structures (empty triangles). Please refer to Methods for the PDB codes of the structures used in this comparison.

**Figure 4:**
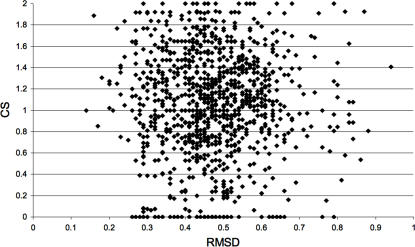
Mapping the relationship between RMSD and centrality in crystallographic conformers. Combined Sensitivity (CS) is plotted against the Root Mean Square Deviation (RMSD) values observed for every pair of structures compared. 31 HIV-1 protease structures in complex with a substrate were compared against 42 HIV-1 protease structures without a substrate. Please refer to Methods for the PDB codes of the structures used in this comparison.

In Figure 3, the fraction of identical central residues shared by every pair of protein conformers (y-axis) was calculated and normalized to 1; so, Figure 3 shows that even when two conformers are similar (e.g., some HIV-1 protease conformers share less than 1 Å RMSD values; see Figure 4 for the RMSD values), their central residues are not the same (no value of 1 was found between any protein conformer compared). To determine if there is a relationship between centrality and the structural differences between the conformers (measured as the Root Mean Square Deviation), we plotted the RMSD against the fraction of central residues shared by every conformer; we found that there is no such relationship (Figure 4).

Thus, we have shown that different protein conformers have different central residues despite the small geometrical differences observed between the proteins and, consequently, that there is no relationship between the overall geometrical differences observed between protein conformers and the occurrence of central residues in these conformers. These results provide the basis to assess axiom **A2**.

### Screening for Protein Functional Conformers

We propose that if a protein conformer participates in a given protein function, it must harbor as central residues those that are critical for that function (axiom **A2**). For instance, protein conformers of an enzyme solved in the presence of its substrate may show as central residues the critical residues involved in binding the substrate. In order to account for this, the sensitivity values reported in the following sections will use as critical residues those critical for ligand binding only, thus differing from the previous results shown so far.

**Figure 5:**
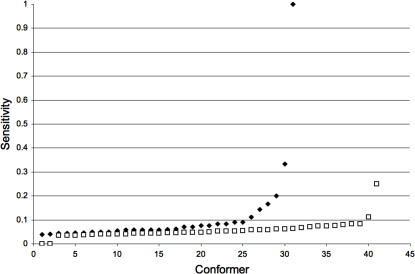
Mapping functional conformers in the HIV-protease by centrality measurements. The overall and average sensitivity for predicting critical residues of the HIV-protease was significantly higher when we used crystallographic structures of the HIV-protease associated with a substrate (black dots) than when the crystallographic structures did not include the substrate (white dots). To facilitate visual analysis, the points of each group were sorted in ascending order according to their sensitivity value.

To evaluate axiom **A2**, we looked at the HIV protease for which there are multiple protein complexes solved with a substrate or an inhibitor. From crystallographic [6] and mutagenesis studies [16], it has been shown that the residues Asp25, Gly27, Asp29, Asp30, Lys46 and Ile50 are critical for substrate binding and/or catalysis. For comparison, we analyzed 42 and 31 HIV protease structures solved in the absence or presence of a substrate analogue, respectively (see Methods for the list of PDB structures). By looking at the fraction of critical residues harbored by these sets of conformers as central residues (expressed as the sensitivity value), we observed that the HIV protease conformers bound to a substrate analogue predominantly show as central residues those that are known to be involved in catalysis (see Figure 5).

**Figure 6:**
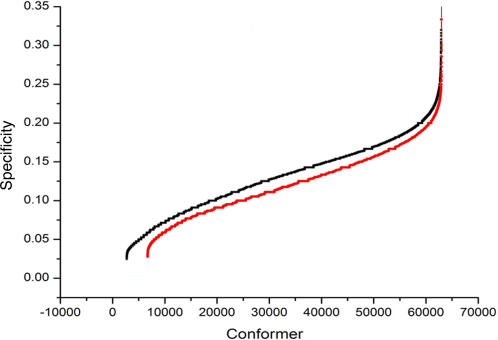
Mapping functional conformers in the TBP by centrality measurements. The overall and average sensitivity for predicting critical residues for the binding of the TBP to the TATA sequence was significantly higher when we used structures derived from a molecular dynamics simulation of the TBP associated with the TATA sequence, (labeled TBP+WtDNA, black dots) than when the simulated structures were without DNA, (labeled TBP, red dots). To facilitate visual analysis, the points of each group (63,000 structures each) were sorted in ascending order according to their sensitivity value. See Table 2 for a statistical analysis of these data.

**Figure 7:**
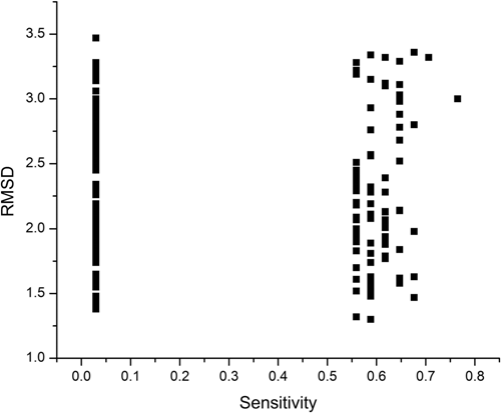
Mapping the relationship between RMSD and centrality in molecular dynamics. TBP conformers with the highest and lowest values of both sensitivity and specificity in the four molecular dynamic simulations of TBP were used to show the relationship between the sensitivity value and the RMSD of the conformer with respect to the 1YTB structure.

We also analyzed multiple computationally generated protein conformers. In these studies, we used the yeast TATA binding protein (TBP), which has been solved both in the presence [18] and in absence [19] of its ligand: the DNA TATA box. It has been previously shown by mutagenesis that at least 53 residues in yeast TBP are involved in DNA binding (see Table 1). We ran four molecular dynamics simulations, and for each of them 63,000 structures were generated. The four simulations included: a) TBP+WtDNA, TBP in the presence of a high affinity substrate (the TATA sequence), using PDB file 1YTB [19] as the starting structure, b) TBP-WtDNA, TBP that was solved in the presence of the TATA sequence (that is 1YTB), but the DNA was not included in the simulation, c) TBP-GCDNA, TBP in the presence of a low affinity substrate (GC sequence) generated by *in silico* substitution of the TATA sequence present in 1YTB by the GCGCGCGCGC DNA duplex and d) TBP solved without substrate, using PDB file 1TBP [18] as a starting structure. The abundance of critical residues for DNA binding found as central residues in these conformers follows the order: a)>b)>c)>d) (see Table 2 and Figure 6). Also, there is no correlation between the RMSD differences of the conformers and the critical residues for DNA binding harbored by these conformers (see Figure 7).

**Table 1:** TBP DNA-Binding Null Mutants.

WT Residue	Mutants	References
Pro65	Ser	[34]
Leu67	Lys	[38]
Asn69	Ser, Arg, deletion	[33],[21],[26],[22],[17]
Val71	Ala, Met, Arg, Glu	[36],[21],[33],[26],[25],[32]
Leu76	Lys	[38]
Leu80	Lys	[38]
Leu82	Lys	[38]
Lys97	Glu	[24]
Arg98	Glu	[21],[22]
Phe99	Lys, Leu	[36],[19]
Ala100	Pro	[27]
Ile103	Lys	[19]
Arg105	Leu, Cys	[36],[19]
Pro109	Ala, Gln	[32]
Lys110	Leu	[38],[39]
Thr111	Ile	[37]
Thr112	Lys	[36]
Ala113	Lys, Leu	[19]
Leu114	Lys, Phe	[38],[19],[21],[31]
Ile115	Lys	[19]
Phe116	Tyr, Lys, Leu	[36],[19],[32]
Ser118	Leu	[18],[29]
Lys120	Leu	[38],[39]
Met121	Lys	[19]
Val122	Arg, Lys	[19],[21]
Thr124	Asn, Arg	[21],[26]
Gly125	Deletion	[26]
Lys127	Leu	[38],[39]
Ser126	Asn	[37]
Arg141	Ala	[38]
Ile143	Asn	[34]
Phe148	Leu	[18]
Lys156	Ala	[38]
Asn159	Asp, Leu, Arg	[23],[18],[21],[29],[32]
Val161	Ala, Glu, Arg	[36],[18],[21],[29],[33],[32]
Leu172	Lys	[38]
Leu175	Lys	[38]
Leu189	Pro, Ser	[28],[20]
Phe190	Arg, Gln, Thr	[20],[21]
Pro191	Ala	[27]
Leu193	Lys	[38]
Ile194	Arg, Phe	[35],[21]
Arg196	Glu, Cys	[36],[21]
Lys201	Glu, Leu	[38],[39],[21]
Val203	Glu, Lys, Thr	[35],[36],[21]
Leu204	Lys	[38]
Leu205	Arg, Val, Lys, Phe	[35],[38],[21],[30],[31]
Phe207	Leu, Tyr	[36]
Lys211	Leu	[38],[39]
Val213	Arg	[21]
Leu214	Lys	[38]
Thr215	Arg	[21]
Lys218	Leu	[38],[39]

WT residue column describes the residue (3-letter code amino acid and its position) in the wild-type TBP that once mutated to any of the amino acids described in the Mutants column, abolished the ability of TBP to bind DNA. The reference numberings reporting such mutants are indicated.

**Table 2:** Statistical Analysis of the TBP Functional Conformer Identification.

Group	N	Mean	SD
TBP+WtDNA	63000	0.254	0.122
TBP-WtDNA	63000	0.249	0.116
TBP-GCDNA	63000	0.234	0.117
TBP	63000	0.23	0.109

Compared Groups	ModelDF	Model MS	Error DF	Error MS	F	α
TBP+WtDNA–TBP-WtDNA	1	0.671	125998	0.014	46.709	0.05
TBP-WtDNA–TBP-GCDNA	1	7.319	125998	0.013	533.58	0.05
TBP-GCDNA–TBP	1	0.564	125998	0.012	43.873	0.05

(Upper Part) Each row shows the statistical parameters for each group of TBP conformers derived from molecular dynamics simulations. TBP+WtDNA: TBP with the TATA sequence. TBP-WtDNA: TBP orginally resolved with the TATA sequence but removed during the simulation. TBP-GCDNA: TBP with a GCGC sequence. TBP: TBP originally resolved without DNA and simulated without DNA. (N: number of conformers, SD: Standard deviation).

(Lower Part) Each row summarizes the results for a one-way ANOVA (Null hypothesis: mean(1st group) = mean(2nd group)) for the pairs of groups indicated in the first column. In each case the null hypotheses is rejected at the 0.05 level of significance (DF: degrees of freedom, MS:mean square, F: Calculated F-value , α: level of significance).

**Figure 8:**
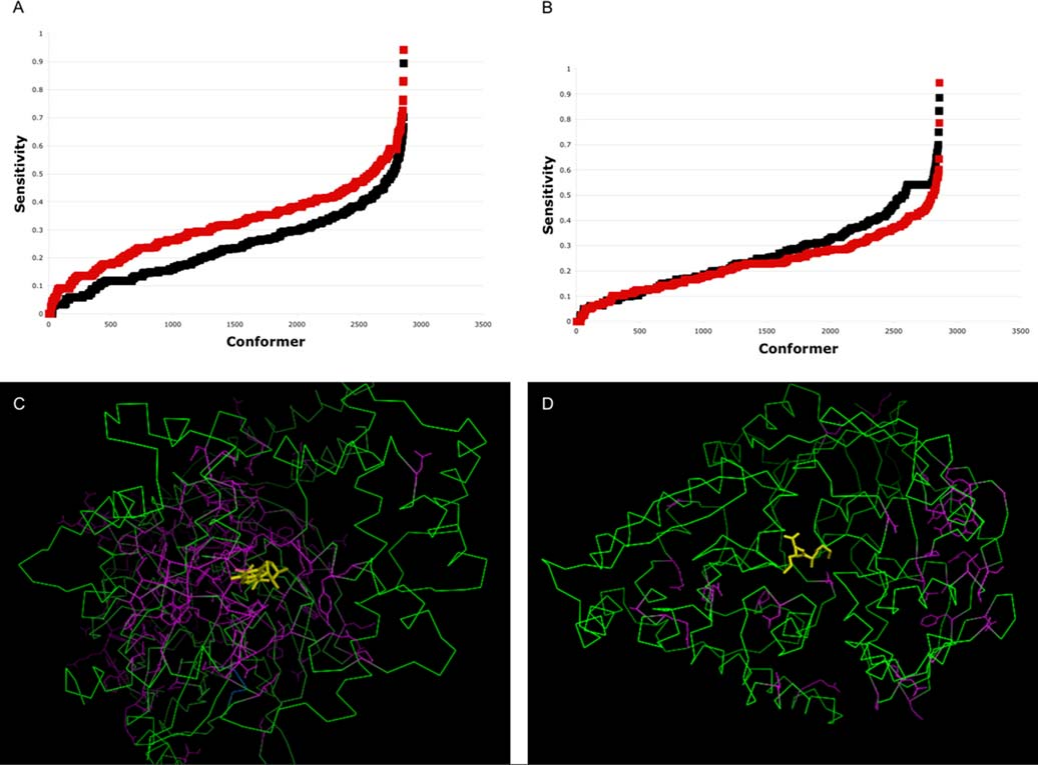
Mapping functional conformers in the MolMov set by centrality measurements. The sensitivity value for predicting critical residues in the MolMov set (see Methods) is plotted against each conformer evaluated. (A) The sensitivity values for 10 proteins with predicted critical residues close to the ligand showed significantly higher values when the protein was associated to a ligand (red squares) than the corresponding protein structures without the ligand (black squares). (B) As in (A), but here 10 proteins are shown for which the predicted critical residues were not close to the ligand. To facilitate visual analysis in (A) and (B), the points of each group were sorted in ascending order according to their sensitivity value. (C) 1CIP, Guanine nucleotide-binding protein in complex with a GTP analogue, is an example of a protein where the predicted critical residues were close to the ligand. (D) 2RKM, Oligopeptide-binding protein in complex with Lys-Lys peptide, is an example of a protein where the predicted critical residues were not close to the ligand. In (C) and (D) the ligand is in yellow, the protein in green, and the critical residues in purple.

In order to analyze the veracity of axiom **A2** and the reliability of our method in a larger data set of proteins, we employed the MolMov set that includes a total of 20 different proteins (see Methods and Table 3). This set includes a subset of protein structures solved in the absence of a ligand (subset U) and a subset of protein structures interacting with a ligand (subset I). A total of 286 alternative conformations were generated for every protein structure in each subset, providing a total of 2,860 protein structures in each subset, as derived from the normal modes of vibration (see Methods). The critical residues for ligand binding for each protein were assumed to be those conserved residues on the protein surface (see Methods). This assumption includes some degree of uncertainty (conserved residues not necessarily are functionally relevant) and provides an additional way to evaluate our procedure (see below). We observed that on average, the proportion of truly predicted critical residues (expressed as sensitivity) in the MolMov subset U is smaller than for the subset I (see Figure 8A) but not in all cases (see Figure 8B). We noticed that the MolMov set included 10 proteins for which the predicted critical residues were closer to the ligand (3 Å on average per protein, data not shown) in the crystal structure (see Figure 8C for an example) than for the other 10 proteins in the MolMov set (see Figure 8D for an example). Thus, only when the critical residues are truly related to the function of interest, our approach can identify the associated conformations to that function. These results are independent of the nature of either the ligand or the protein analyzed (see Table 3).

**Table 3:** The MolMov Set.

PDB Code	Protein Name	Ligand Name	SCOP Classification
1BJY	Tetracyclin repressor	Tetracycline	All alpha
1DQY	Antigen85c (mycolyltransferase)	Diethyl phosphate inhibitor	Alpha/beta
1CRX	CRE recombinase	DNA	All alpha
1EX7	Guanylate kinase	Guanosine 5-monophosphate	All beta
1QUK	Phosphate-binding protein	Phosphate	Alpha/beta
1GTR	Glutaminyl-tRNA synthase	ATP	Alpha/beta
2DRI	Ribose-binding protein	Ribose	Alpha/beta
1SSP	Uracyl-DNA glycosylase	Uracyl-DNA	Alpha/beta
1CIP	Guanine nucleotide-bindign protein	Phosphoaminophosphonic acid-guanylate ester	Alpha/beta
3PJR	Helicase	DNA	Alpha/beta
1B0O	Beta-Lactoglobulin	Palmitate	All beta
6TIM	TriosePhosphate Isomerase	3-Phosphoglycerol	Alpha/beta
1F8A	Peptidyl-prolyl cis-trans isomerase	Phosphoserine-proline peptide	Alpha + beta
1DVJ	Orotinide monophosphate dehydrogenase	6-AZA Uridine MonoPhosphate	Alpha/beta
1FTM	Glutamate receptor	AMPA	Alpha/beta
3MBP	Maltose-binding protein	Maltotriose	Alpha/beta
1QAI	Reverse transcriptase	Nucleic acid	Multidomain protein (alpha and beta)
2RKM	Oligopeptide binding protein	Lys-Lys peptide	Alpha/beta
1I7D	DNA toposiomerase II	8-bases single-stranded DNA	Multidomain protein (alpha and beta)
1PFK	Phosphofructokinase	Fructose diphosphate	Alpha/beta

The proteins solved in complex with a ligand in the MolMov set are listed with their ligands. The first ten rows correspond to the protein whose predicted critical residues were close to the ligand; the last ten rows are the proteins whose predicted critical residues were not close to the ligand. The last column indicates the structural classification as indicated in the SCOP database.

### Linking Mutagenesis Data to Protein Structure and Dynamics

The 53 mutants listed in Table 1 were identified with TBP-DNA binding gel-shift assays [20]–[42]. The assay does not distinguish between folding-defective mutants and mutants directly involved in DNA binding. In contrast to the HIV protease, there are not numerous structures of the yeast TBP bound to the TATA DNA, thus limiting our ability to establish the structure/dynamics/function relationship of these mutants. For instance, the assumption that only residues less than 5 Å from DNA are directly involved in binding eliminates residues that are at a longer distance from DNA; yet, these distant residues may be at 5 Å or closer to the DNA in some alternative conformations of TBP bound to DNA. If multiple protein structures are computationally generated to determine which residues always fall within a cut-off distance from DNA, there is no a priori knowledge to determine if all possible conformations were explored. Thus, simply measuring the distance between the ligand and the protein does not provide a comprehensive method to link structure to biological function. Similar reasoning may be applied to energy calculations, since there is no a priori energy value that may be used to specify the relevant residues for binding. In this context, our method does not measure the distance between the ligand and protein, thus is complementary to the criteria based on the distance between the ligand and a protein and could be used to improve our ability to identify critical residues for protein-ligand interactions.

All 53 critical residues in TBP involved in DNA binding qualified as central residues in the structures generated during the simulations (see Table 4). This indicates that the simulations sampled relevant conformations of TBP associated to the function of the 53 DNA-binding null mutants. However, the centrality criteria used to map critical residues onto protein structures does not distinguish between critical residues for structure and binding. Thus, we examined if there are differences in the presence of these critical residues in the simulations. We would expect that critical residues found exclusively in simulations of TBP in the presence of DNA are more likely to be involved in binding, while those residues prevalently found in all the simulations (frequency> = 0.50) are more likely to be involved in maintaining TBP structure. From Table 4, we identified Lys97, Ser118, Pro191, Lys211, Val213 and Thr215 (yeast TBP numbering) as residues critical for binding, whereas critical residues for TBP structure would be Leu67, Leu76, Leu80, Val122, Leu172 and Leu175. In agreement with the yeast TBP-DNA structure, all residues that were predicted to be involved in DNA binding are oriented towards it, while those predicted to be involved in TBP structure actually are in the protein's core, with the exception of Val122, which faces DNA. Moreover, Leu67, Leu76, Leu80, Leu172 and Leu175 were shown to produce misfolded proteins upon mutation to Lysine [18].

**Table 4:** Observed Frequencies of Critical Residues for DNA Binding Found as Central Residues in Simulated Conformers of TBP.

WT Residue	TBP+WtDNA	TBP-WtDNA	TBP	TBP-GCDNA
Pro65	0.579	0.277	0.247	0.429
Leu67	0.786	0.621	0.552	0.57
Asn69	0.237	0.051	0.045	0.026
Val71	0.268	0.016	0.015	0.015
Leu76	0.842	0.911	0.81	0.689
Leu80	0.897	0.969	0.861	0.728
Leu82	0.435	0.286	0.255	0.161
Lys97	0.001	0	0	0
Arg98	0.026	0.034	0.03	0
Phe99	0.233	0.294	0.261	0.098
Ala100	0	0.044	0.039	0.003
Ile103	0.045	0.022	0.019	0.037
Arg105	0.102	0.013	0.011	0.02
Pro109	0.075	0.044	0.04	0.057
Lys110	0.029	0.017	0.015	0.034
Thr111	0.279	0.124	0.11	0.208
Thr112	0.433	0.551	0.49	0.287
Ala113	0.049	0.029	0.025	0.098
Leu114	0.578	0.652	0.579	0.21
Ile115	0.35	0.551	0.49	0.422
Phe116	0.184	0.244	0.217	0.389
Ser118	0.001	0	0	0
Lys120	0.344	0.496	0.441	0.421
Met121	0.326	0.089	0.079	0.128
Val122	0.665	0.961	0.854	0.569
Thr124	0.11	0.042	0.037	0
Gly125	0.025	0.032	0.029	0.01
Lys127	0.338	0.273	0.243	0.274
Ser136	0.221	0.122	0.109	0.103
Arg141	0.014	0.001	0.001	0
Ile143	0.168	0.132	0.117	0.075
Phe148	0.164	0.071	0.063	0.078
Lys156	0.213	0.156	0.139	0.223
Asn159	0.323	0.343	0.305	0.287
Val161	0.247	0.221	0.197	0.141
Leu172	0.897	0.998	0.887	0.758
Leu175	0.958	1.007	0.895	0.757
Leu189	0	0.033	0.029	0
Phe190	0.046	0.03	0.027	0
Pro191	0.001	0	0	0
Leu193	0.479	0.497	0.442	0.336
Ile194	0.016	0.016	0.015	0.002
Arg196	0.045	0.035	0.031	0.031
Lys201	0.003	0.003	0.002	0.082
Val203	0.063	0.023	0.02	0.048
Leu204	0.087	0.053	0.048	0.061
Leu205	0.071	0.033	0.03	0.023
Phe207	0.054	0.001	0.001	0.002
Lys211	0.049	0	0	0
Val213	0.093	0	0	0
Leu214	0.453	0.336	0.299	0.245
Thr215	0.013	0	0	0
Lys218	0.572	0.644	0.572	0.477

The observed frequencies of DNA-binding null mutant positions (WT residue) for each of the 4 molecular simulations, including: a) TBP+WtDNA, b) TBP-WtDNA, c) TBP and d) TBP-GCDNA. The frequencies were obtained by normalizing the number of times any of the residues in this table was detected as central in all of the 63,000 conformations analyzed.

## Discussion

Under the current view that proteins accomplish their function through a set of conformations [4],[43], we postulate that the known critical residues play their roles in that set of conformations. In such a case, having a method to map critical residues to protein structures will assist in the identification of the protein conformations associated to the function of the critical residues. In previous reports, it has been shown that central residues to protein structure are related to residues critical for protein function (e.g., folding, catalysis) [11]–[15]. In all these previous studies, central residues have been detected in a single protein structure. However, protein function comprises an ensemble of protein structures and presumably, each protein structure may harbor a different subset of central and critical residues. Supporting this notion, Vendruscolo and cols. [14] showed that central residues in the folding transition state of 6 proteins map only critical residues for folding. Along this line, we showed that in the folded states of 131 proteins, central residues map to critical residues for either keeping the structure and/or binding [11],[12]. Here, we show additional evidence that including multiple conformers of a given protein improves the relationship observed between central residues and critical residues for protein function in three different proteins (see Figures 1 and 2). Taken together, axiom **A1** is supported by these results.

Note that simply including many protein conformers in the analysis may not identify more critical residues. In this case, it is important to take into account the diversity of conformations being analyzed and the mechanism used by the protein to recognize the ligand (e.g., induced-fit versus selected-fit mechanisms. See below).

Additionally, our results are in agreement with the notion that conserved residues are not always functionally important, yet some conserved residues have functional roles (e.g., catalytic residues). Also, our results indicate that different protein conformers may harbor different central residues and, presumably different functions (axiom **A2**). If such is the case, our goal to identify functional conformers computationally seems reachable.

Indeed, we show that different protein conformers harbor different sets of central residues (see Figure 3), despite their structural similarities (<1 Å) as measured by RMSD (see Figure 4). Consequently, we found that there is no relation between the difference in central residues in different conformers and the geometrical differences, measured as RMSD, amongst the conformers (see Figure 4 and 7), indicating that centrality is not simply a measure of the geometrical differences between protein structures. Thus, the data indicate that central residues seem to be fingerprints of protein conformations.

Understanding this correspondence between centrality and protein structure may lead to generate protein structures hosting specific sets of critical and central residues. This will require a more in-depth characterization of the topological features of protein structures represented as networks. Recognizing our current limitation to generate protein conformers harboring a specific set of central residues, our best approximation to identify functional conformers of proteins is through the screening of collections of protein structures.

We determined the central residues for 73 experimentally determined conformers of the HIV protease and for 252,000 computationally generated conformers of TBP. For these two proteins, the critical residues for binding the substrate or other ligand have been identified [6], [16], [20]–[42]. It is important to note, that it may be possible to have more than a single protein conformer binding a substrate/ligand, provided also that the substrate/ligand exists in several conformations. Given this condition, it is not surprising to find several conformers of these two proteins harboring as central residues those matching the critical residues for binding the substrate/ligand (see Figures 5 and 6). As expected, the protein conformers harboring most of the central residues corresponding to the critical residues for binding the substrate/ligand, are the experimentally determined conformers bound to the substrate/ligand (see Figures 5 and 6). We observed a similar trend for a larger data set of 20 different proteins (see Figure 8A). However, the protein structures in complex with a ligand cannot be identified if the critical residues provided are not related to the binding of such ligand (in our case, derived from a conservation index of exposed residues; see Figure 8B and 8D). These results are independent of the nature of either the ligand or the protein analyzed (see Methods and Table 3).

Thus, according to axiom **A2** critical residues for ligand binding will exert their function in the protein conformers bound to the ligand. In terms of our procedure, axiom **A2** implies that central residues found in protein conformers bound to the ligand will include mostly the critical residues for ligand binding. Our results provide support to this axiom and provide the feature to identify the functional conformers of proteins.

We noticed that some conformers derived from the protein structure in the absence of a ligand actually present large sensitivity values (see Figures 5, 6, and 8A). Understanding these results will require further studies, but a possible explanation could be found in the mechanism of action used by the proteins to recognize their ligands. For instance, in the induced-fit mechanism [4],[5], proteins in the absence of a ligand, will rarely adopt a conformation observed when proteins are bound to the ligand, and only when the ligand is present such conformations will be frequently observed. According to our postulate, a protein conformation in the absence of a ligand will harbor less frequently central residues matching critical residues. That is the case for the HIV protease (see Figure 5); however, the yeast TBP dynamics shows a large number of conformers in the absence of a ligand with a high proportion of central residues matching critical residues for binding (see Figure 6). This suggests a possible induced-fit mechanism for the HIV-1 protease but not for the yeast TBP. Our results then, could be interpreted according to the mechanism used by the protein to recognize its ligand. However, further studies will be required to validate the usefulness of our approach in determining the mechanism of protein interactions and are out of the scope of the current work.

To illustrate the usefulness of our method in the study of the structure/dynamics/function relationship of proteins, we examined previously reported mutants of the yeast TBP that have been identified as critical for DNA binding. Since binding to DNA is a dynamic process, it is important to keep in mind that a single structure of TBP in complex with DNA may not be sufficient to determine which of the residues have a role in binding or in keeping the structure. We explored the use of our method for distinguishing these residues. Our results show that residues Lys97, Ser118, Pro191, Lys211, Val213 and Thr215 are more likely involved in binding, while residues Leu67, Leu76, Leu80, Val122, Leu172 and Leu175 appeared to be involved in the preservation of the structure of yeast TBP. It is important to note that our method does not use a criterion based on the distance of the protein to the ligand; nonetheless our results are in consonance with the distance and orientation of the critical residues observed in the structure of yeast TBP in complex with the TATA-box DNA. Likewise, mutations on the residues predicted to be involved in maintaining TBP structure (Leu67, Leu76, Leu80, Leu172 and Leu175) do not transcribe in either an activated (in the presence of transcription activators) or basal fashion, supporting the idea of a structural role for these residues [18]. Interestingly, residue Val122 is predicted to be involved in maintaining TBP structure but it faces DNA, suggesting that Val122 may have a dual role: DNA binding and structure maintenance. Further experimental evidence is required to elucidate this possibility.

### Conclusions

Our results support the notion that protein function is achieved through an ensemble of protein conformations [4],[43]. The method shown here may be applied to any other protein of interest to identify its potential functional conformers. For that purpose, we have made available the software to identify central residues at http://bis.ifc.unam.mx/jamming/ [12]. The identification of functional conformers of a target protein is indeed useful in many different areas of research, such as drug design, protein function design and protein-protein interaction predictions, among others. Likewise and as shown here, the ability to map critical residues onto protein structures may increase our capacity to link experimental data with structural information. For instance, in many mutagenesis studies of proteins, especially those that test the in vivo function of the mutants, it is not obvious if the defects in function are related to a folding and/or processing problem, or to a more subtle functional effect. Our method may aid in the interpretation of such data.

## Materials and Methods

### Data

To study the relationship between conserved residues and central residues in multiple protein structures, two proteins were used: HIV protease and the T4 lysozyme. For the HIV protease, 73 experimentally determined crystal structures were used: 1a30, 1a8g, 1a9m, 1aaq, 1ajv, 1ajx, 1axa, 1bdr, 1bv7, 1bv9, 1bwa, 1bwb, 1cpi, 1dif, 1dmp, 1gnm, 1gnn, 1gno, 1hbv, 1hih, 1hiv, 1hos, 1hps, 1hpv, 1hpx, 1hsg, 1hte, 1htf, 1htg, 1hvc, 1hvi, 1hvj, 1hvk, 1hvl, 1hvr, 1hvs, 1hwr, 1hxb, 1hxw, 1mer, 1mes, 1met, 1meu, 1mtr, 1odw, 1odx, 1ody, 1ohr, 1pro, 1qbr, 1qbs, 1qbt, 1qbu, 1sbg, 1tcx, 1vij, 1vik, 1ytg, 1yth, 2aid, 2bpv, 2bpw, 2bpx, 2bpy, 2bpz, 2upj, 3aid, 4hvp, 4phv, 5hvp, 7hvp, 8hvp, 9hvp. For the T4 lysozyme 23 experimentally determined crystal structures were used: 1ctw, 1cu0, 1cu2, 1cu3, 1cu5, 1cu6, 1cup, 1cuq, 1cv0, 1cv1, 1cv3, 1cv4, 1cv5, 1cv6, 1cvk, 1cx7, 1d2w, 1d2y, 1d3f, 1d3j, 1d3m, 1d3n, 1qsq.

To identify functional conformers three sets of protein structures were used: HIV protease, the yeast TATA-Binding Protein (TBP) and the MolMov set of proteins. For the HIV protease, the same protein structures described above were used. The PDB code of those structures in complex with a substrate analogue are: 1aaq, 1cpi, 1dmp, 1hbv, 1hih, 1hiv, 1hos, 1hps, 1hpv, 1hte, 1htf, 1htg, 1hvi, 1hvj, 1hvk, 1hvl, 1hvr, 1hvs, 1ohr, 1sbg, 2bpv, 2bpw, 2bpx, 2bpy, 2bpz, 4hvp, 4phv, 5hvp, 7hvp, 8hvp, 9hvp. For TBP, the crystal structures used had the PDB codes: 1tbp for TBP without DNA, and 1ytb for the TBP complex with a TATA box (TATATAAA).

In the case of the MolMov set, we used the proteins reported at the database of macromolecular movements [44]. This database includes structures of proteins motions and we have analyzed only those including an interaction with a ligand. Thus, this set includes protein structures in the absence of a ligand (MolMov subset U) and the structures of the same protein solved in the presence of a ligand (MolMov subset I). The PDB codes in the MolMov subset U includes: 1bjz, 1beb, 1dqz, 1tre, 1pin, 1dv7, 4crx, 1ex6, 1fto, 1omp, 1rkm, 1oib, 1nyl, 1urp, 1akz, 1d6m, 1gp2, 2pfk and 1pjr. The PDB codes in the MolMov subset I include: 1bjy, 1b0o, 1dqy, 6tim, 1f8a, 1dvj, 1crx, 1ex7, 1ftm, 3mbp, 1qai, 2rkm, 1quk, 1gtr, 2dri, 1ssp, 1i7d, 1cip, 1pfk and 3pjr. 10 of these proteins showed the predicted critical residues close to the ligand, while the other proteins showed the predicted critical residues not so close to the ligand (see Table 3). The MolMov set includes very diverse types of ligands and protein architectures (see Table 3) and the number of amino acids per protein ranked from 156 to 647. Finally, for each structure in these subsets, 26 normal modes of vibration were calculated using ElNèmo [45] and 11 protein conformations derived for each. Thus, the MolMov set includes a total of 5,720 protein structures, with 2,860 protein structures in each subset.

### TBP Molecular Dynamics

The initial structure for the simulation of free TBP was 1TBP [18] (PDB code). The structure 1YTB [19] (chains B and D), which is the carboxyl terminal domain of TBP from *Saccharomyces cerevisiae* bound to a TATA box hairpin (5′ TATATAAA 3′, CYC1), was used as the initial structure; the bases in the hairpin were removed, and only 10 basepairs were kept (the TATA box and one-basepair at the 5′ and 3′ end). The complex of TBP bound to sequence 5′ GCGCGCGCGC 3′ (CG) was constructed introducing the necessary modifications to the 1YTB structure using the Biopolymer module of InsightII program. The structures were solvated placing the solute molecules on a cubic TIP3 water box and removing all the waters within 2.5 Å of the solute. The cubic water box was trimmed to a hexagonal box employing the Simulaid program [46]. Initially, the water molecules and sodium atoms were submitted to an energy minimization using 4 stages of 500 Steepest Descent (SD) steps and 2 stages of 1000 Adopted Basis Newton-Raphson (ABNR) steps. After solvent minimization, periodic boundary conditions (PBC) were turned on employing the CRYSTAL module of the CHARMM [47] program version 28 using CHARMM27 parameters [48],[49]. The solvent was again minimized with 500 ABNR steps keeping the solute molecule fixed. Two final minimization stages were applied to the whole system with 250 SD steps and 250 ABNR steps. The solvent was equilibrated with 150 ps of molecular dynamics using a 1.5 fs step in the NPT ensemble at 300 K with the Leap-Frog integrator. Later, the whole system was equilibrated using the same protocol for the solvent. The Berendsen algorithm was used. A value of 600.0 atomic mass units (amu) was used for the mass of the pressure piston. The reference pressure was set to 1 atm. The Langevin piston collision frequency was set to 10.0 ps^−1^. The Langevin piston bath temperature was set to 300 K. The Hoover constant temperature was used. The Hoover reference temperature was set to 300.0 K. The mass of the thermal piston was set at 1000 kcal*ps^−2^. The target temperature was 300 K. The image and neighbor list update were done when necessary (heuristic test), with a distance cut-off set to 14 Å; electrostatic interactions were shifted, and van der Waals interactions were switched, to ensure smooth forces at the cutoff distance. All calculations were performed using SHAKE algorithm and an integration time step of 1.5 fs was used. All the systems were simulated for 10.65 ns using PBC with the CRYSTAL module of CHARMM in the NPT ensemble at 300 K with the Leap-Frog integrator saving coordinates every 100 steps. The last 9 ns were used for analysis.

### Building Networks and Identifying Central Residues from Protein Structures

Networks were derived from protein structures by a distance criterion. That is, two residues were considered neighbors and consequently to interact if at least 1 atom on each residue is 5 Angstrom (Å) apart or closer. The atoms within that distance may be part of the amino acid's main chain and the amino acid's side chain. Therefore, the networks that were built had amino acid residues as nodes and their interactions as links. Links were labeled with identical weights. We previously reported that among 21 different ways to build networks from protein structures (e.g., distance between center of masses, charge, different distance cut-off values), this way reproduces with better results the prediction of critical residues from central ones [12]. Central residues were defined as those residues with the largest transitivity values having the same frequency in the network (see Figure S1 for an example). The transitivity values were obtained by counting the number of times a residue was in the shortest paths connecting every pair of residues in the network. The frequency of a transitivity value is the number of residues presenting that transitivity value in a network. Thus, each residue will have a transitivity value and a frequency in the network; only those having transitivity values immediately close to the largest transitivity value in the network and with the same frequency as those with the largest transitivity values are considered central. Using this strategy we observe that about 20% or less of the residues were central given a protein structure (see Figure S1 for an example). For these calculations, we used our software available at http://bis.ifc.unam.mx/jamming/ [12]. Transitivity, T, is related to betweeness, B as follows: Bi = Ti/SPi; where Bi is the betweeness value calculated for the i-node, Ti is the Transitivity value of the i-node, and SPi is the number of shortest paths connecting the i-node to the rest of the nodes in the network.

### Estimating the Reliability of the Predictions

Two measurements were used to account for this: sensitivity and specificity. Sensitivity, Se, is defined as Se = (TP+FN)/AP, where TP: true positives, FN: false negatives and AP: all positives. In our case, AP are all the critical residues determined experimentally, TP are the critical residues correctly predicted and FN the critical residues not predicted as critical. Specificity, Sp, is defined as Sp = (AN−FP)/AN; where AN: all negatives and FP: false positives. In our case, AN are the non-critical residues determined experimentally and FP are the residues predicted as critical, which are not critical. Additionally, in order to compare the sensitivity of the predictions in paired comparisons (see Figure 4), we defined the Combined Sensitivity parameter as:
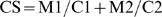
Where C1 refers to the observed central residues in protein 1 and, C2 refers to the observed central residues in protein 2. M is the number of central residues that are truly critical residues for either protein 1 or protein 2. Thus, 2< = CS> = 0 to distinguish it from Sensitivity.

### Prediction of Critical Residues as Conserved Residues

The *ConSurf* server [50] was used for this. The parameters used to run the *ConSurf* server were: Maximum likelihood method used to calculate the conservation scores, PSI-BLAST E-value = 0.001, maximum number of homologous sequences = 50 and the number of PSI-BLAST iterations = 1. Conserved residues were those with the most negative score (color code of 9).

## Supporting Information

Figure S1 Transitivity distribution The transitivity values (Y-axis) obtained for each residue (X-axis) in the yeast TATA-Binding Protein (1TBP, chain B) are shown as rhombs. The values are ordered by transitivity value to facilitate the visual analysis of the data. The central residues are the most traversed residues that present the same frequency, and are presented as filled rhombs on the top right corner. That is, there are 6 residues with the largest transitivity value of 17 (Tyr139, Met121, Phe227, Ile212, Ile160, Leu175); the next lower transitivity value is 16 and also presents the same frequency (6 residues: Ile143, Val123, Ile70, Leu76, Ile223, Leu214) than those with transitivity value of 17; similarly there are 6 residues with transitivity value of 15 (Ile115, Ser136, Met104, Ile170, Leu234, Ile206). Note that residues with transitivity value of 14 have a frequency different than 6 and thus were not considered as central. Only the 18 residues with transitivity values of 17, 16, and 15 are considered central to the 1TBP structure.
(0.08 MB TIF)
