# Supplementary figures and images for: Computer-Based Screening of Functional Conformers of Proteins
Source: PLoS Comput Biol. 2008 Feb 29;4(2):e1000009. doi: 10.1371/journal.pcbi.1000009 (PMC2265533; doi:10.1371/journal.pcbi.1000009)

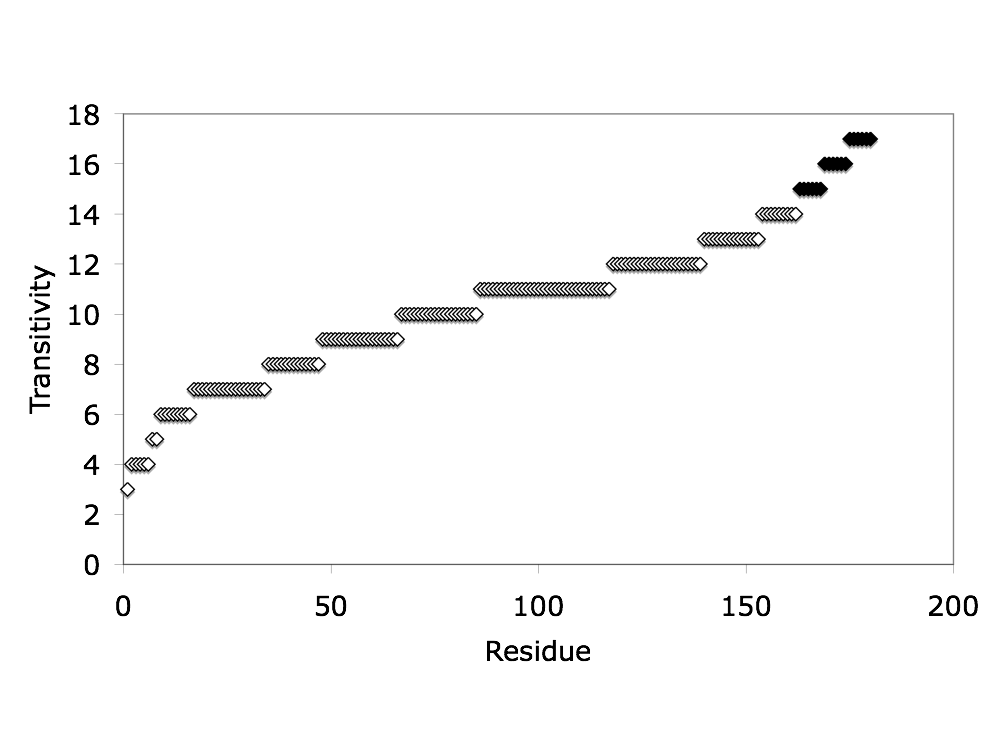

Supplement: Figure S1 Transitivity distribution — The transitivity values (Y-axis) obtained for each residue (X-axis) in the yeast TATA-Binding Protein (1TBP, chain B) are shown as rhombs. The values are ordered by transitivity value to facilitate the visual analysis of the data. The central residues are the most traversed residues that present the same frequency, and are presented as filled rhombs on the top right corner. That is, there are 6 residues with the largest transitivity value of 17 (Tyr139, Met121, Phe227, Ile212, Ile160, Leu175); the next lower transitivity value is 16 and also presents the same frequency (6 residues: Ile143, Val123, Ile70, Leu76, Ile223, Leu214) than those with transitivity value of 17; similarly there are 6 residues with transitivity value of 15 (Ile115, Ser136, Met104, Ile170, Leu234, Ile206). Note that residues with transitivity value of 14 have a frequency different than 6 and thus were not considered as central. Only the 18 residues with transitivity values of 17, 16, and 15 are considered central to the 1TBP structure. (0.08 MB TIF) [file pcbi.1000009.s001.tif]
